# Augmenting Epidemiological Models with Point-Of-Care Diagnostics Data

**DOI:** 10.1371/journal.pone.0153769

**Published:** 2016-04-20

**Authors:** Özgür Özmen, Laura L. Pullum, Arvind Ramanathan, James J. Nutaro

**Affiliations:** 1 Computational Science and Engineering Division, Oak Ridge National Laboratory, Oak Ridge, Tennessee, United States of America; 2 Health Data Sciences Institute, Oak Ridge National Laboratory, Oak Ridge, Tennessee, United States of America; Shanxi University, CHINA

## Abstract

Although adoption of newer Point-of-Care (POC) diagnostics is increasing, there is a significant challenge using POC diagnostics data to improve epidemiological models. In this work, we propose a method to process zip-code level POC datasets and apply these processed data to calibrate an epidemiological model. We specifically develop a calibration algorithm using simulated annealing and calibrate a parsimonious equation-based model of modified Susceptible-Infected-Recovered (SIR) dynamics. The results show that parsimonious models are remarkably effective in predicting the dynamics observed in the number of infected patients and our calibration algorithm is sufficiently capable of predicting peak loads observed in POC diagnostics data while staying within reasonable and empirical parameter ranges reported in the literature. Additionally, we explore the future use of the calibrated values by testing the correlation between peak load and population density from Census data. Our results show that linearity assumptions for the relationships among various factors can be misleading, therefore further data sources and analysis are needed to identify relationships between additional parameters and existing calibrated ones. Calibration approaches such as ours can determine the values of newly added parameters along with existing ones and enable policy-makers to make better multi-scale decisions.

## Introduction

Influenza-like illnesses (ILI) are key contributors to mortality rates within the United States (US). In spite of extensive surveillance of patients (both inpatients and outpatients) for flu-like symptoms, the reporting lag in most surveillance measures remains between 2 and 4 weeks, which makes it difficult to adopt effective strategies for limiting disease spread and prevention. However, traditional surveillance still relies on the use of clinical and laboratory tests for assessing the spread of the disease. Point-of-care (POC) diagnostics for ILI surveillance have been adopted within the US, especially in the context of emerging influenza pandemics. These diagnostics include reports of hospital/laboratory confirmed diagnostics, viral culture and sub-typing, all of which are reported to county-wide, state-wide, and nation-wide agencies. POC datasets are widely used as a high-resolution and trusted measure of ILI prevalence. They are aggregated and reported to public health officials at the national and regional scales, and made available to the public via various websites and public health applications available on mobile phones and other devices. The seasonal variations (accrued since 2001) encoded by this aggregated data are used by the Centers for Disease Control and Prevention (CDC) and other agencies to guide public health policies in matters of determining vulnerable populations, assessing preparedness, and deciding which populations must be inoculated/quarantined in case of a sudden outbreak.

The use of epidemiological models, including equation-based models (EBM) and agent-based models (ABM), is also becoming common for gaining insights into disease spread across different scales (regional to national) and to guide policy decisions about intervention strategies. The utility of these models can be enhanced if POC diagnostics data (of observations) can be used to calibrate the simulation process. Recently, we developed three model variations (one EBM and two ABM models—one with ADEVS [1] and one using RePAST [2]) of the same SIR dynamics [3]. We used 1918 influenza epidemic parameter values derived from the literature as input into these models to explore the impacts of modeling assumptions. This study uses the simplest possible model (*Delayed-SIR* from [3]) to understand if the outputs from the model can produce results similar to the observed POC diagnostics data. We aim to answer the following question: *Do the simple Epidemiology models predict the observed infections documented in POC diagnostics data*?

In this work, three steps are performed to calibrate the model: (i) we acquired distinct POC datasets at zip-code level, (ii) we proposed a method to process POC datasets for meaningful comparison against SIR models, and (iii) we employed a global optimization meta-heuristic (*Simulated Annealing*) to develop a calibration algorithm and calibrate the model. Our results show that parsimonious SIR models are remarkably capable of predicting peak loads, and that calibration yields parameter values similar to those reported in the literature.

Our analysis also revealed the quantitative limitations (i.e., predicting onset of the epidemic) of the simple model. Additional research is needed to address these limitations and to guide decisions on what parameters and processes should be added to the simple model. As a first step in this direction, we tested an intuitive hypothesis that population members of more densely populated areas encounter greater numbers of people, and this in turn results in greater peak loads. Our results show that even simple intuitive assumptions are subject to reconsideration when the data is rich, and further data and analysis are needed to parameterize and calibrate extensions to the simple model.

## Materials and Methods

We acquired data from four distinct data sources: (1) POC diagnostics data for 2009–2010 at zip-code level for the whole US (POC-Data), (2) 2010 Census Data (Census), (3) POC diagnostics data collected from the state of Tennessee Knox County providers during the 2009 H1N1 influenza outbreak (Knox-Data), and (4) POC diagnostics data collected from the state of Texas for the 2009 H1N1 influenza outbreak (Texas-Data). In the following section, we describe the data processing method and calibration algorithm.

### Data Processing Method for POC-Data

The POC-Data includes the number of daily H1N1 observations between April 1 (2009) and March 31(2010) from 14,098 distinct zip-codes throughout the US. Our future goal is to fuse the POC-Data with the Census data, so that we can explore and extract patterns that can be used for the validation of complex simulation studies. However, Census data provides geographic information using ZCTA (Zip-code Tabulated Area) information instead of zip-codes as the smallest geographic unit because a zip-code can correspond to a mailbox or a building that does not have any residents. One ZCTA can span multiple zip-codes, while a zip-code can also belong to multiple ZCTAs (See [4] for further guidance on the relationship between zip-codes and ZCTAs). Of the 14,098 zip-codes represented in the POC-Data, 10,518 zip-codes can be mapped to Census ZCTAs. Then, of these 10,518 zip-codes, we considered only those ZCTAs (8,994 ZCTAs) that span a single zip-code, because the POC-Data does not include H1N1 observations for all constituent zip-codes of each ZCTA.

In general, two outbreaks are observed during the H1N1 season: (i) Spring and (ii) Autumn. Since the Autumn outbreak was more intense, we focused on the zip-codes exhibiting an Autumn outbreak. The Autumn outbreak is assumed to start mid-July [5] and we observed the data until late-January (a 200-day window). We considered only the zip-codes that observed more than 5 incidents in the Autumn. Consequently, our processed POC-Data consisted of 7,632 zip-codes that are distributed over 50 states.

There was a bias introduced by the data collection process (i.e., most of the POC-Data is entered on Thursdays). Therefore, we employed moving averages (of 7 days) to aggregate the data. Also, reported cases in any POC dataset are likely to under-estimate the true impact of an epidemic. It is challenging to estimate the true numbers, because many people with influenza do not seek medical care, only a small number of people who seek care are tested, and hospitalizations and deaths are under-reported as well. Hence, we used the proportion of total infectious population over time for calibration purposes. This metric is formed by normalizing the number of newly infected people at each day by the total number of observed infections during the 200-day window. The equation below explains how the proportion of infectious is calculated:

$$\begin{array}{c} PI_{i}=\frac{OI_{i}}{\sum_{i=1}^{N}OI_{i}} \end{array}$$ (1)

where *PI*_*i*_ is the proportion of the total population who are infectious at day *i*, *OI*_*i*_ is the number of observed infections at day *i*, and *N* is the total number of days evaluated (200 in this case).

### Computational Data

In SIR models, the population is divided into three compartments: Susceptible (S), Infectious (I) and Recovered (R). Although our implementation of the model allowed us to add an incubation period, we specifically considered the simplest possible model (*Delayed-SIR*) due to its modest need for computational resources, while retaining its ability to generate known trajectories of the number of people at each compartment over time. When the incubation period is added, the model becomes a Susceptible-Exposed-Infectious-Recovered (S-E-I-R) model, which is a more granular version of the SIR model. It would only add an additional delay to the point where we observe the peak in the epidemic, so we preferred to keep the complexity the simplest and did not add the incubation period.

In the *Delayed-SIR* model, the dynamics of the disease spread process is captured by the interactions between the compartments, modeled as deterministic ordinary differential equations (ODE). The model is developed using the MODELICA programming language and solved using the OpenModelica solver (*www*.*openmodelica*.*org*). The model also considers discontinuities that occur at the early stages of simulation runs (i.e., to account for the recoveries of the initially infected persons). There is no death process in the model and the *R* population is assumed to preserve immunity that prevents them from becoming sick again. The equations for the *Delayed-SIR* model are as follows:

$$\begin{array}{c} \frac{d}{dt}S=-pt\times I\times \frac{S}{S+I+R}\\ \frac{d}{dt}I=-\frac{d}{dt}S-\frac{d}{dt}R\\ \frac{d}{dt}R=\left\{\begin{array}{cc} I(t-tr) & \text{if}\;t>tr\\ 0 & \text{otherwise} \end{array}\right. \end{array}$$ (2)

The simulation model uses input values derived from online resources and literature for the non-calibrated parameter values. The definitions of parameters and their corresponding values are as follows:

1. *Persons to infect (pt)*: Number of persons one infectious person infects per day (unit = person/day). This value is *calibrated*.
2. *Time to Recovery (tr)*: Time to recover from the disease (unit = day). It is assumed to be 7 days based on [6].
3. *Initial Infectious (IInf)*: Number of individuals who are initially infectious (unit = person). It is *calibrated*. *I* = *IInf* at day 0.
4. *Total Population (tp)*: Total number of people in the simulation population (unit = person). It is assumed to be 50,000, which will allow the disease to spread among the population. It only impacts the magnitudes of the outputs and does not have a significant impact on the trajectory of model outputs [7]. *S* = *tp*—*IInf* at day 0.

Regarding the output metrics, traditional SIR models monitor the number of people at each compartment over time. For meaningful comparison against a POC dataset, the number of newly infected people on each day is output from the simulation runs. This output is also aggregated using moving averages (of 7 days). Then, this aggregated data is normalized by the total number of infections observed during the simulation run, which forms the *proportion of infectious* as in Eq 1.

### Calibration Algorithm

Three metrics are particularly important for policy-makers while evaluating the transient dynamics of an epidemic and planning intervention strategies. These metrics are *peak load* on health services, defined as the greatest number of infectious persons observed at any time; *diffusion speed*, defined as the amount of time that passes from the initial outbreak until the peak load is observed; and *total burden* defined as the cumulative number of people who died from the disease until the epidemic vanishes from the population. In this work, we only focus on *peak load* and *diffusion speed* since there is no death process in the model.

We use a global optimization algorithm (Simulated Annealing [8]) to calibrate the simulation model against the POC-Data for each zip-code separately. Simulated annealing (SA) is a local search algorithm that starts from an initial solution, then generates a neighbor to that solution. A fitness function (cost) is calculated and if there is a reduction in cost (in the case of a minimization problem) then the existing solution is replaced with the generated neighbor solution. This procedure is continued until no improvement is observed or a certain number of iterations is reached. The calibration procedure is shown in Algorithm 1 (See [9] and [10] for details).

**Algorithm 1** Calibration Algorithm Pseudo-code

Set the number of iterations *it* to terminate;

Set termination tolerance *tol*;

Select an initial temperature *T* > 0;

Set the temperature (*T*) coefficient *α*;

Select the initial state *x*;

Run the simulation and process outputs;

Calculate the cost *σ*;

Set best cost *λ* to *σ*;

Set iteration counter *n* = 0;

**while** *λ* > *tol* and *n* < *it* **do**

 Generate state *i*, a neighbor of *x*;

 Run the simulation using *i* and process outputs;

 Calculate cost *σ*;

 **if** *σ* < *λ* **then**

  Set *x* = *i* and *λ* = *σ*;

 **else if** *random*(0, 1)<*exp*(−(*σ* − *λ*)/*T*) **then**

  Set *x* = *i* and *λ* = *σ*;

 **else**

  do nothing;

 **else if**

 Set *n* = *n*+1;

 Set *T* = *T*×*α*;

**end while**

Algorithm 1 is tested against two cost (fitness) functions measuring: (i) Diffusion Speed and (ii) Peak Load. The first cost function is interested in shifting the curve on x-axis (time) by altering *IInf*:

$$\begin{array}{c} Minimize\frac{\left|v_{d}-v_{s}\right|}{v_{d}} \end{array}$$ (3)

where *v*_*d*_ is diffusion speed observed in POC-Data and *v*_*s*_ is diffusion speed generated by a simulation run. The second cost function is interested in fitting the height of the curve (y-axis magnitude) by altering *pt*, where *p*_*d*_ is the peak load observed in POC-Data and *p*_*s*_ is the peak load generated by simulation runs.

$$\begin{array}{c} Minimize\frac{\left|p_{d}-p_{s}\right|}{p_{d}} \end{array}$$ (4)

**Fig 1:**
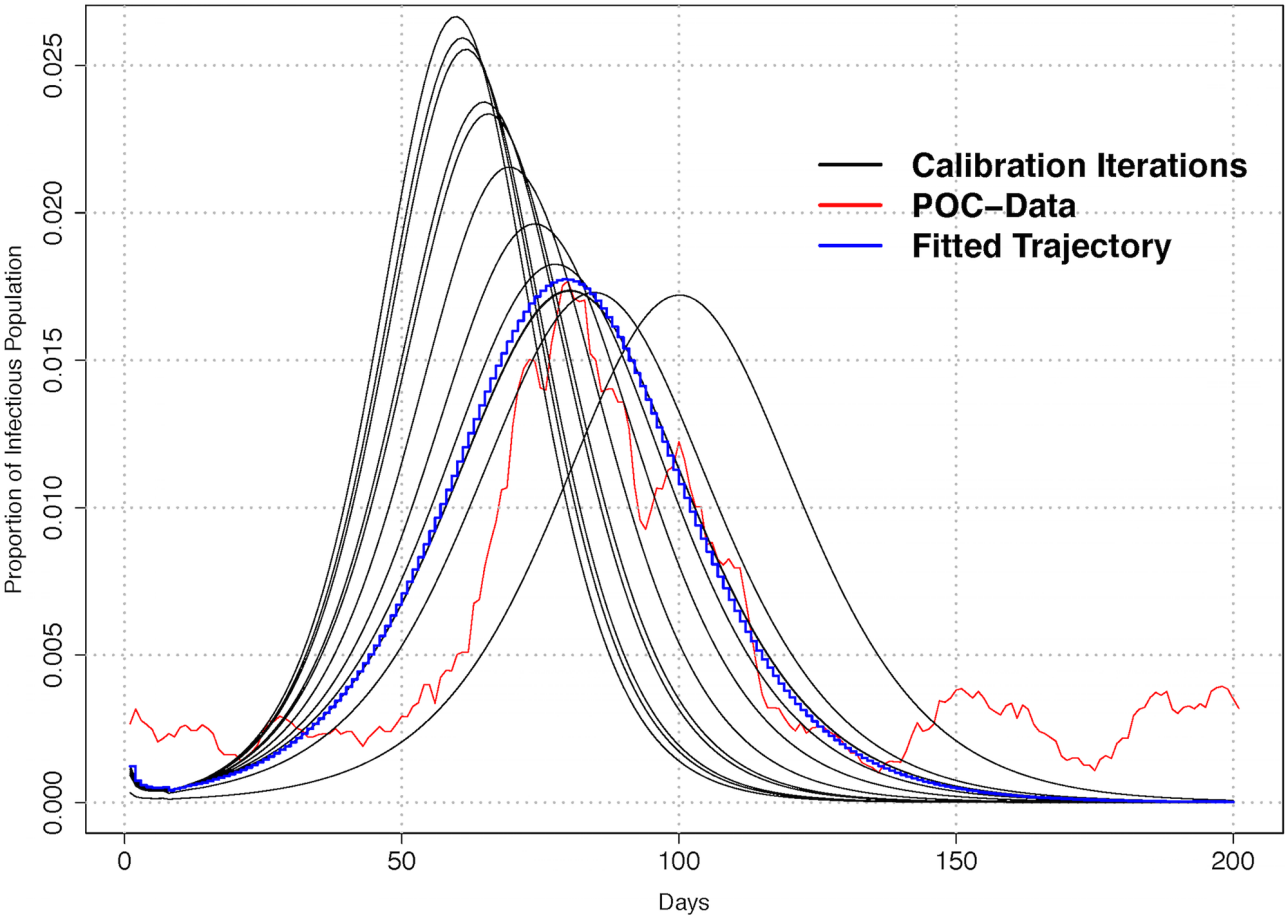
Sample convergence of simulation iterations to POC-Data.

We use the calibration algorithm to fit Eq 3 first, fix *IInf*, then continue with fitting Eq 4. We conduct these steps for each zip-code in the data. Each time the algorithm is called, it runs until a conservative number of iterations (i.e., *it* = 25) are completed or until the fitness value is lower than the tolerance (i.e., *tol* = 0.005). For faster convergence, *α* of temperature *T* is set to 0.90 and the moving operator (which generates neighbors) guides the direction of the search based on what side of the data points the simulation output falls. Fig 1 illustrates the convergence of simulation runs (iterations) to the POC-Data values. It can be perceived from Fig 1 that output trajectories of simulation iterations shift on the *x* and *y* axes until a good match against POC-Data is observed.

## Results

### Fitness Performance of the Calibration Algorithm

**Fig 2:**
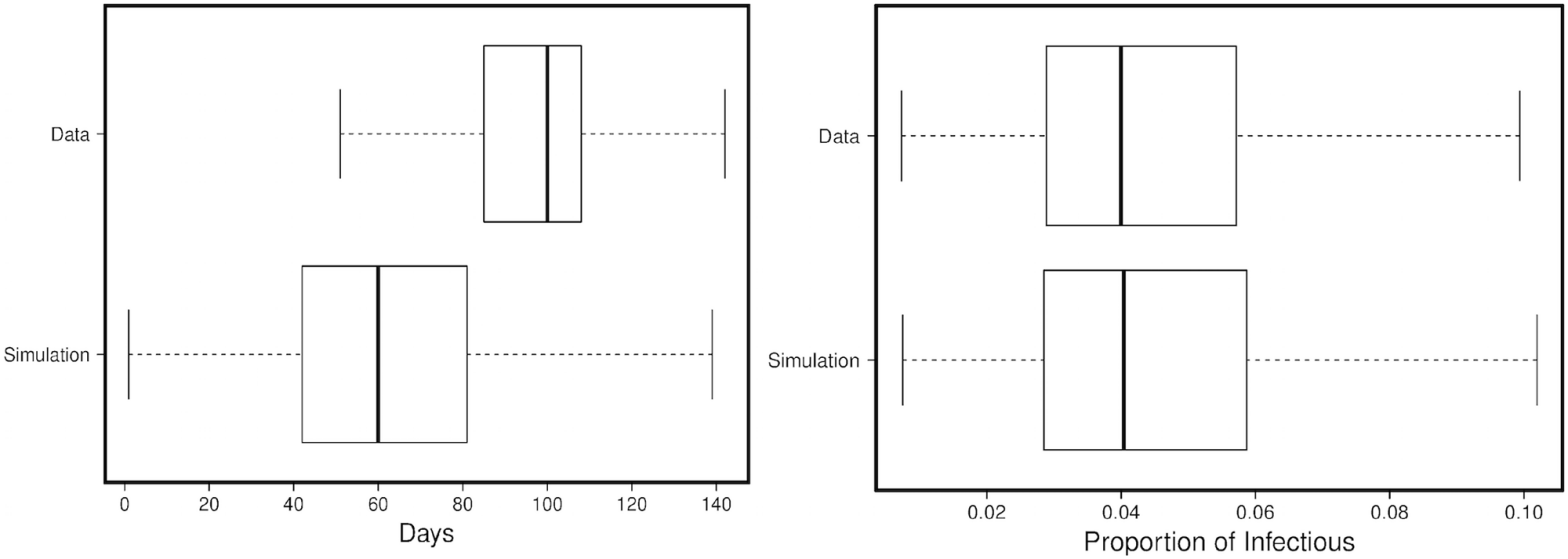
The distribution of diffusion speed (on the left) and peak load (on the right) for all zip-codes.

Fig 2(a) and 2(b) illustrate the performance of the calibration algorithm. As observed in Fig 2(a), although we shift the curve by altering *IInf*, its impact is limited. As expected, onset of the outbreak occurs earlier in the simulation runs than in the POC-Data. This is due to the model assumption that the initial infectious population starts to spread the disease at day zero, while the actual onset of the disease-spread could be based on numerous factors (i.e., geographic location, population dynamics, and weather conditions). Fig 2(b) illustrates the close fit between the POC-Data and the simulation runs for the peak load.

Table 1 summarizes the fitness of Eq 4 after it is applied to all zip-codes. Considering that we have not done an exhaustive search and interrupted the calibration algorithm after a relatively small number of iterations, the performance is quite promising.

**Table 1:** Summary statistics of Eq 4 cost function values for all zip-codes.

Minimum	1st Quartile	Median	Mean	3rd Quartile	Maximum
0.000000	0.001502	0.003158	0.069440	0.004690	4.840000

### Validation of Calibrated Reproduction Rate

*R*_0_ is the reproduction rate that determines the number of secondary infections an infected person generates assuming that the population is fully susceptible [11]. It serves as a threshold and depends on two parameters in SIR models: (i) *pt*, and (ii) *tr*[12]. It has to be greater than one for the disease to propagate.

$$\begin{array}{c} R_{0}=pt\times tr \end{array}$$ (5)

**Fig 3:**
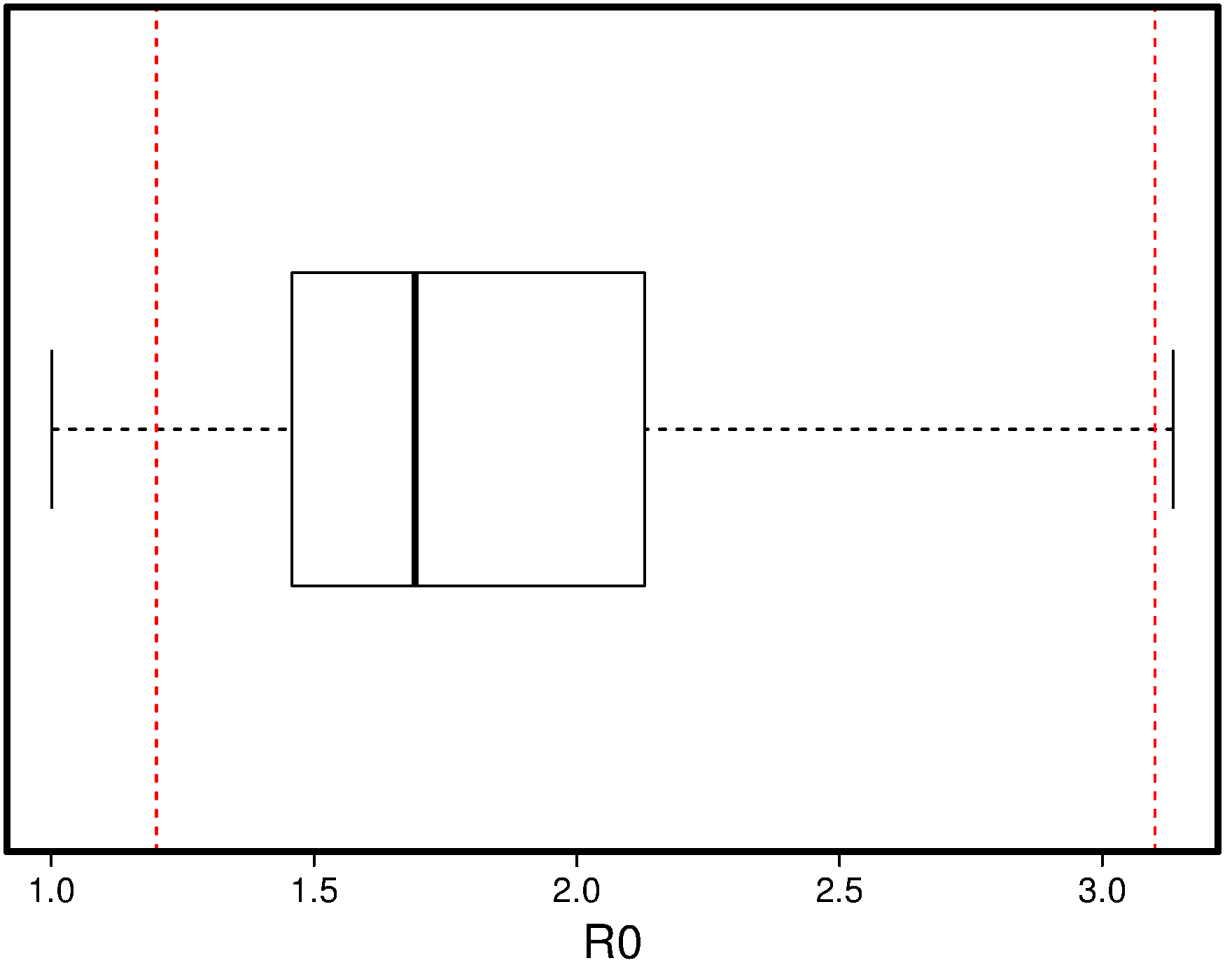
The distribution of *R*_0_ values found by calibration (Red lines represent the range in the literature).

Based on studies that investigated the early stages of the outbreak in the US, the H1N1 outbreak in 2009–2010 had *R*_0_ values ranging between 1.3–3.1 [13–15]. Although the average number of secondary cases is likely to be lower than the *R*_0_ estimates and is definitely lower than our calculation in the simulation results, it serves as a threshold with the assumption of a fully susceptible population. Fig 3 illustrates the distribution of *R*_0_ values of the calibrated *pt* values in the early stages of the disease. Calibrated values are within the *R*_0_ ranges empirically supported in the literature.

### Calibration against Distinct Datasets

In this section, we present calibration results of the algorithm against two distinct datasets: (1) Texas-Data and (2) Knox-Data. Texas-Data is collected by the Texas State Department of State Health Services (DSHS) as part of its H1N1 influenza surveillance program. This data represents a validated data source for understanding the recorded number of infected patients with respect to the H1N1 influenza outbreak in 2009 (April 1, 2009–March 31, 2010). Texas-Data consists of age, zip-code, and date information along with the information on whether collected specimens are tested as H1N1 positive. The dataset includes information from 40 different zip-codes in the state of Texas. Due to the limited number of daily observations and the lack of data for the Autumn outbreak, we focused on the Spring outbreak in Texas-Data during a 200 day window (starting April 1, 2009). Additionally, as in the previous analysis, the moving average (of 7 days) method is employed and the proportion of infectious is compared.

**Fig 4:**
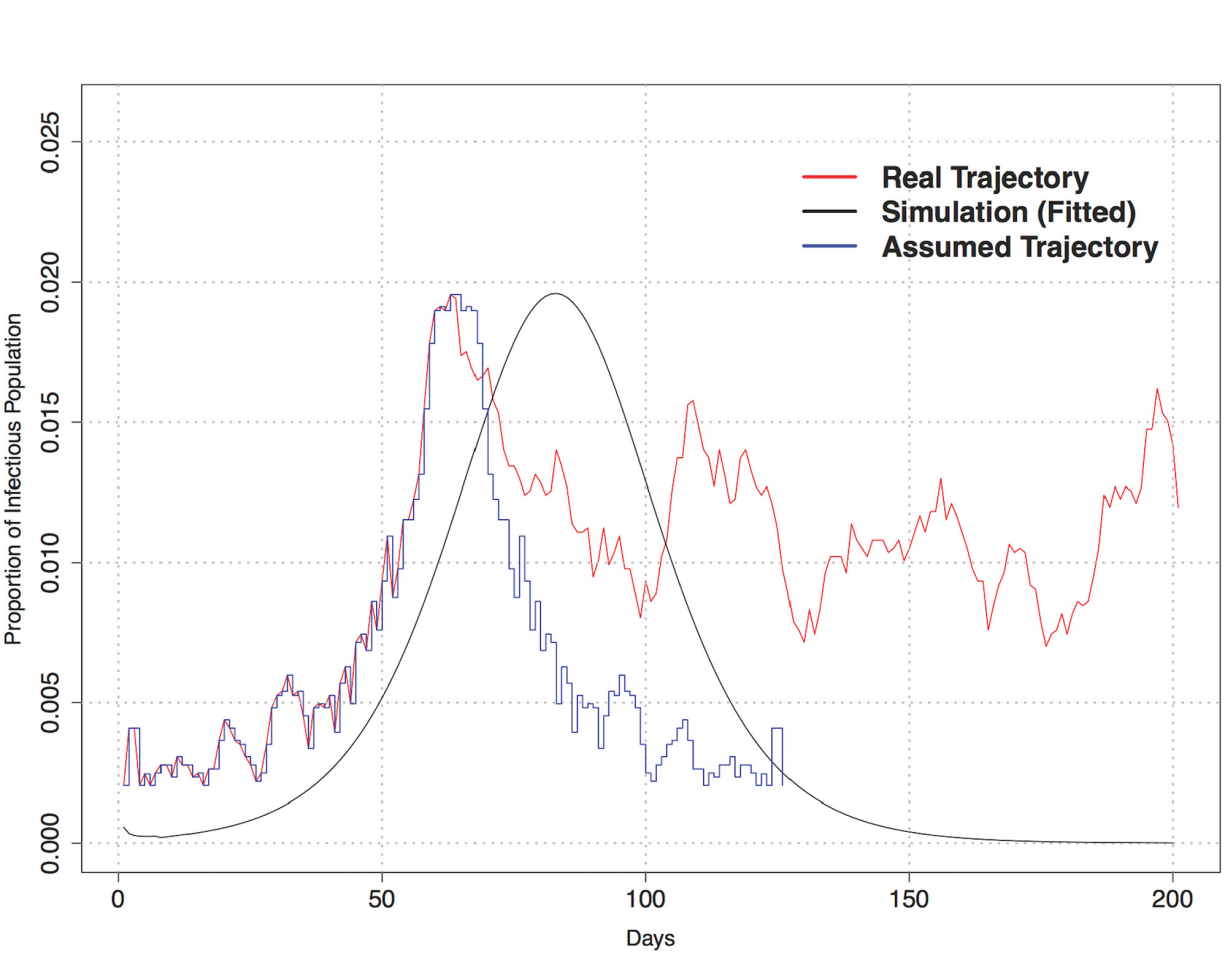
Trajectory of proportion of infectious—Simulation vs. Knox-Data.

Knox-Data is collected from providers in 31 distinct zip-codes that are framed within Knox County in Tennessee. This data source presents number of cases with certain symptoms (i.e., abdominal pain, chest pain, pneumonia, diarrhea, nausea and vomiting, sore throat) during the H1N1 pandemic year (April 1, 2009–March 31, 2010). We assumed that all patient arrivals that reported these symptoms had one of a variety of possible ILI. However, this assumption generates a time-series of the number of infectious population that begins by oscillating for the first ∼ 40 days (starting July 1), then increases to the H1N1 peak load, then around day 80, again oscillates this time at a level greater than the tail of a typical SIR curve. This behavior is different than the observations in the POC-Data and this observation indicates the impact of other seasonal influenza viruses on the reported levels of ILI in the Knox-Data. Therefore, to eliminate the impact of seasonal viruses and illnesses on the levels observed in the Knox-Data, we assume that the peak load that follows the initial oscillations are only caused by the spur of the H1N1 outbreak in that particular zip-code. Next, we calibrate the simulation runs against this assumed dataset. Fig 4 illustrates the data aggregation for Knox-Data.

In the Texas-Data, 15 zip-codes that exhibit more than 50 H1N1 incidents throughout the year are selected for calibration. The Knox-Data includes 31 zip-codes used in calibration. We matched these zip-codes in the Texas-Data and the Knox-Data with the ones in the POC-Data to form POC-Knox and POC-Texas datasets. The POC-Knox data has information for 9 zip-codes out of 31 zip-codes in the Knox-Data. The POC-Texas data has information for 7 zip-codes out of 15 zip-codes in the Texas-Data.

**Fig 5:**
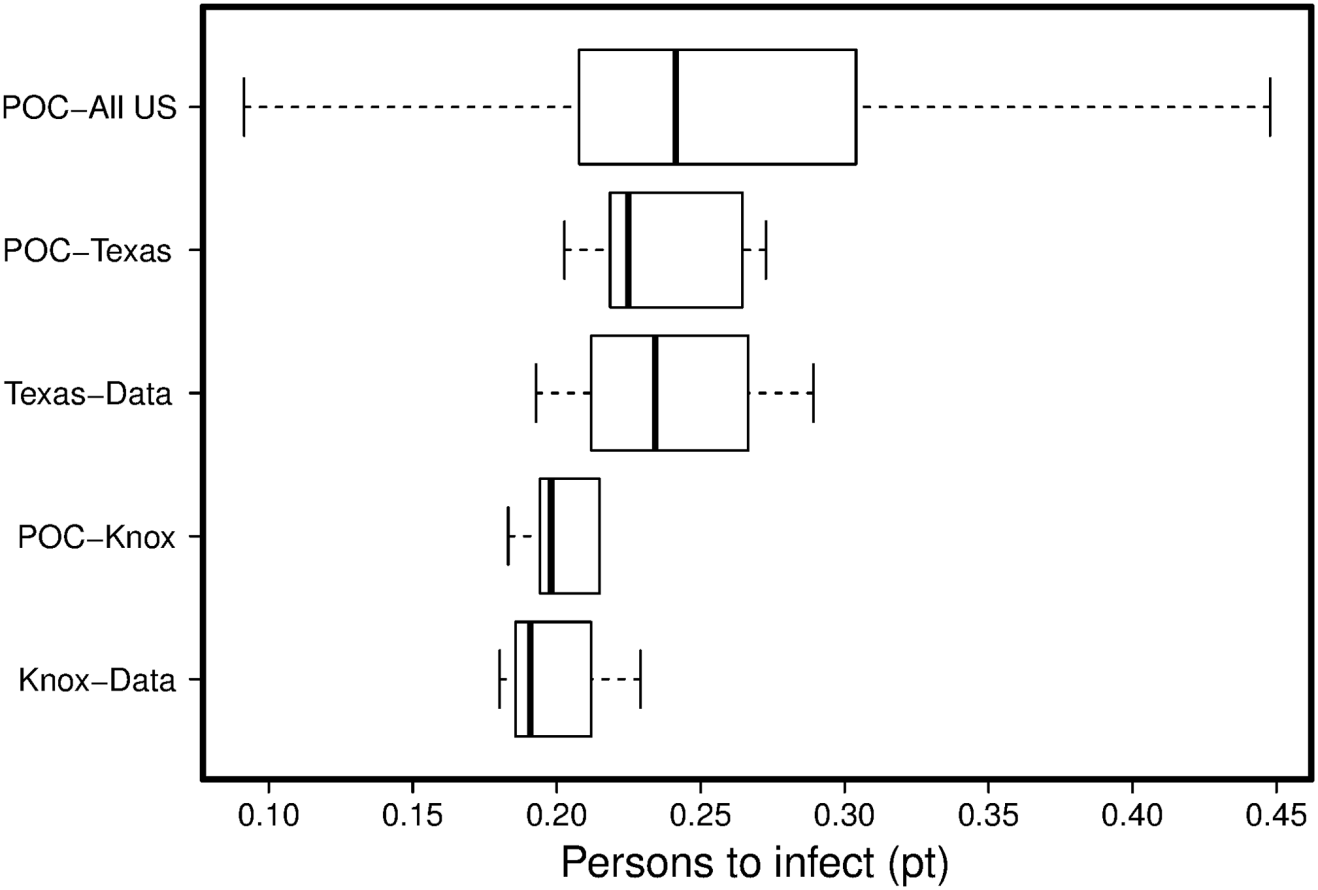
Comparisons of calibrated *pt* values against distinct datasets.

Subsequently, we ran the calibration algorithm optimizing Eqs 3 and 4 consecutively. The calibration algorithm was run against Knox-Data, POC-Knox, Texas-Data, and POC-Texas zip-codes, separately. Fig 5 illustrates distributions of calibrated *pt* values. Convergence to similar levels of *pt* against distinct datasets in the same areas (i.e., Knox-Data vs. POC-Knox) further verifies the capability of our calibration algorithm.

### Potential use of Calibration Algorithm

Finally, we compare density information for each ZCTA (population per square mile using the Census) against the peak loads of POC-Data and simulation results. Intuitively, more dense population areas are likely to exhibit larger outbreaks (as measured by numbers of infectious), which should be reflected in the peak load. Assuming that all other factors are the same, we tested this hypothesis by calculating the Spearman correlation coefficients (*rho*) presented in Table 2. We also present actual numbers in comparison to the proportion of infectious.

**Table 2:** Spearman correlation coefficients during AUTUMN outbreak.

Relationship	rho	p-value
Density vs. Peak Load (Actual)	0.1437582	< 2.2e-16
Density vs. Peak Load (Proportion)	−0.2699924	< 2.2e-16
Density vs. Peak Load (Calibrated Simulation)	−0.2664335	< 2.2e-16

While actual numbers of infectious positively correlate with the density as anticipated by our hypothesis, the proportion of infectious is negatively correlated. This change in direction of correlation suggests that: (i) the relationship between peak load and density is non-linear or (ii) there are other factors (i.e., spatial information, vaccination, data collection bias) that alter the results, or (iii) both are true. Table 2 further verifies that the calibration algorithm is capable of predicting the observed negative correlation between peak load (as proportion of infectious) and density.

**Fig 6:**
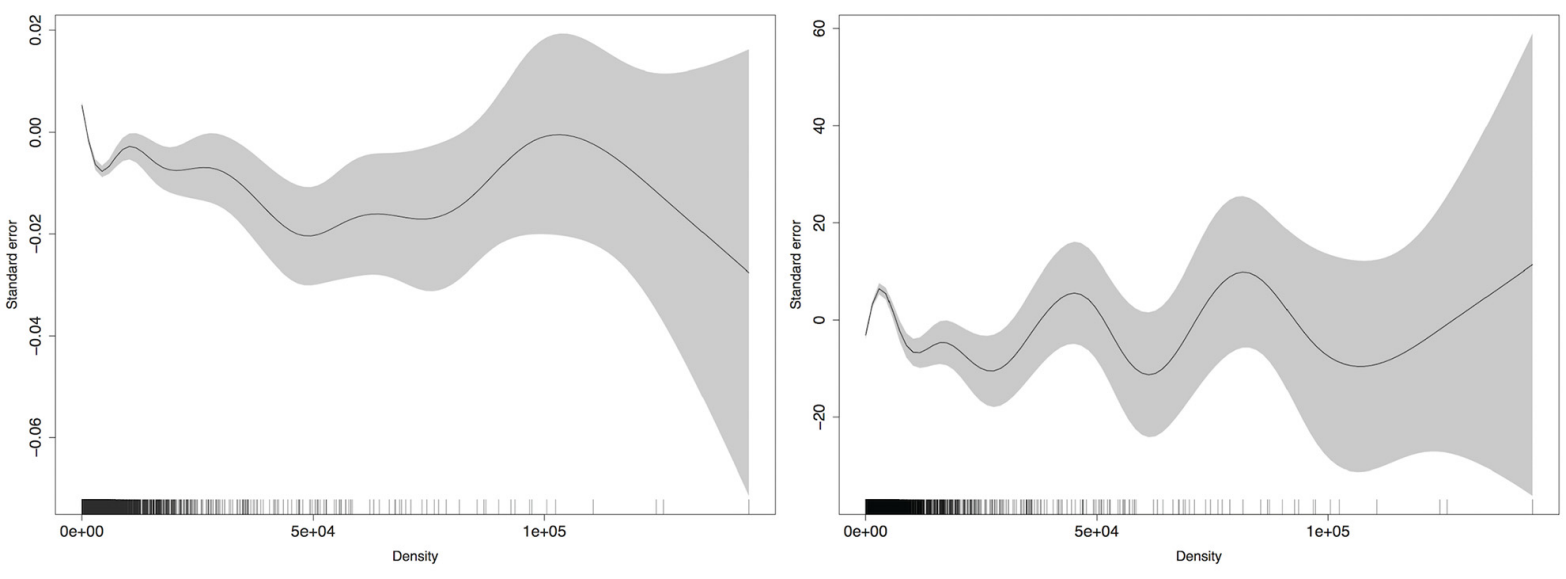
GAM predictions—Density vs. Deviation from the mean Peak Load (Proportion of Infectious) on the left and mean Peak Load (Actual) on the right.

To test non-linearity, we used the generalized additive models (GAM [16]) method to fit a smoothing function using density to the POC-Data (peak load ∼ *s*(density)) and observed its evolution. Fig 6 represents the default plot of GAM results. Solid lines represent predicted values of peak load as a function of density. Small vertical lines on the *x* axis show the location of the sample points. The gray area is interpreted as the standard errors of the estimates provided by the fitted function. As expected, the fitted functions exhibit changes of direction that are indicative of possible non-linear relationships between the density of the population and the peak load. This also questions the linearity assumption in the correlation comparison.

## Discussion

In conclusion, we show that parsimonious models are remarkably effective in predicting the dynamics observed in the number of infected people and in general, the model agrees with the observations from POC diagnostics data. First, we selected an EBM (*Delayed-SIR*) that mimics SIR dynamics to calibrate using the observations [3]. Subsequently, we obtained a rich POC dataset that covers the US to employ in calibration of the selected model. We proposed a method to process POC datasets for calibration purposes. Calibration provides a set of parameter ranges that can be tested against validated data studies and be used in future scenario exploration. We show that a simple meta-heuristic such as *simulated annealing* is sufficiently capable of predicting peak loads observed in POC diagnostics data while staying within reasonable and empirical parameter ranges (*pt* and *R*_0_) reported in the literature. We also tested the calibration algorithm against distinct data sources to further verify the capability of our calibration approach.

Our method can be utilized on any compartmental epidemiological model. However, depending on the disease specific dynamics and the number of parameters to calibrate, the fitness functions should be re-evaluated and the number of iterations in the calibration algorithm should be re-visited. As a future venue of research, if a user wants to employ an agent-based model with spatial characteristics, our calibration algorithm can evolve its parameters and peak loads can be compared against data. In our analysis, each simulation run mimics a single zip-code area. Let us assume that the model with spatial characteristics mimics the epidemic for the entire US and we compare the outputs of individual zip-codes against the POC diagnostics data. Then we need to define more complex fitness metrics to minimize (i.e., summation of mean squared errors between the model output and data for each zip-code). We believe that more sophisticated optimization methods should be explored due to computational time constraints if zip-code specific dynamics are explored via a spatial model for the US. The *Delayed-SIR* model takes 1–2 seconds per run while a more complex model with spatial aspects which runs the epidemic over the entire US would need an extensive amount of computational time per run. Additionally, those models are likely to be stochastic. They need multiple replications for each scenario, which would significantly increase the required computational time.

Following the calibration studies, a logical question arises: *How can the calibrated values be used in future studies?* If the aim of such a study is to estimate the intensity of transmission in a zip-code when a similar outbreak occurs, then our calibration algorithm would give insights on the zip-code specific expectations. But it does not explain why transmission rates occurred at that level in a specific zip-code. Another use-case would arise when further data sources and analyses are available to identify relationships between additional parameters and existing calibrated ones. This necessity is evident when we compare POC-Data and calibration results against Census *density* data per zip-code. Although, intuition and social interaction theories suggest specifying a direction of causation between variables to be able to model a real phenomenon as accurately as possible, linearity can be an oversimplified assumption. Future research is needed to merge new data sources such as demographic distributions (age, gender, etc.), genomic data, and vaccination data at the zip-code level to test relationships against POC diagnosis datasets. Testing several relationships can ultimately help us to identify causal directions among different variables that can further be added as parameters/mechanisms to SIR models. Our calibration algorithm can calibrate these new parameters against the POC-data that is fused with newly augmented additional information. These more complex models and calibrated values can be leveraged to determine the best and worst case scenarios for particular zip-codes when a new pathogen arrives. This capability would help policy-makers gain insights into zip-code specific disease dynamics.
